# Accuracy and reproducibility of fast fractional flow reserve computation from invasive coronary angiography

**DOI:** 10.1007/s10554-017-1190-3

**Published:** 2017-06-22

**Authors:** A. R. van Rosendael, G. Koning, A. C. Dimitriu-Leen, J. M. Smit, J. M. Montero-Cabezas, F. van der Kley, J. W. Jukema, J. H. C. Reiber, J. J. Bax, A. J. H. A. Scholte

**Affiliations:** 1 Department of Cardiology, Leiden University Medical Center, Albinusdreef 2, Postal zone 2300 RC, 2333 ZA Leiden, The Netherlands; 2 Medis Medical Imaging Systems B.V., Leiden, The Netherlands; 3 Department of Radiology, Leiden University Medical Center, Leiden, The Netherlands

**Keywords:** Fractional flow reserve, Computational fluid dynamics, Quantitative coronary angiography

## Abstract

Fractional flow reserve (FFR) guided percutaneous coronary intervention (PCI) is associated with favourable outcome compared with revascularization based on angiographic stenosis severity alone. The feasibility of the new image-based quantitative flow ratio (QFR) assessed from 3D quantitative coronary angiography (QCA) and thrombolysis in myocardial infarction (TIMI) frame count using three different flow models has been reported recently. The aim of the current study was to assess the accuracy, and in particular, the reproducibility of these three QFR techniques when compared with invasive FFR. QFR was derived (1) from adenosine induced hyperaemic coronary angiography images (adenosine-flow QFR [aQFR]), (2) from non-hyperemic images (contrast-flow QFR [cQFR]) and (3) using a fixed empiric hyperaemic flow [fixed-flow QFR (fQFR)]. The three QFR values were calculated in 17 patients who prospectively underwent invasive FFR measurement in 20 vessels. Two independent observers performed the QFR analyses. Mean difference, standard deviation and 95% limits of agreement (LOA) between invasive FFR and aQFR, cQFR and fQFR for observer 1 were: 0.01 ± 0.04 (95% LOA: −0.07; 0.10), 0.01 ± 0.05 (95% LOA: −0.08; 0.10), 0.01 ± 0.04 (95% LOA: −0.06; 0.08) and for observer 2: 0.00 ± 0.03 (95% LOA: −0.06; 0.07), −0.01 ± 0.03 (95% LOA: −0.07; 0.05), 0.00 ± 0.03 (95% LOA: −0.06; 0.05). Values between the 2 observers were (to assess reproducibility) for aQFR: 0.01 ± 0.04 (95% LOA: −0.07; 0.09), for cQFR: 0.02 ± 0.04 (95% LOA: −0.06; 0.09) and for fQFR: 0.01 ± 0.05 (95% LOA: −0.07; 0.10). In a small number of patients we showed good accuracy of three QFR techniques (aQFR, cQFR and fQFR) to predict invasive FFR. Furthermore, good inter-observer agreement of the QFR values was observed between two independent observers.

## Introduction

Current practice directives recommend the use of fractional flow reserve (FFR) measurement to guide revascularization with a class I level A indication according to the European Society of Cardiology guideline on myocardial revascularization [1]. FFR is defined as the ratio of the mean distal coronary pressure to the mean aortic pressure during maximum hyperaemia, usually induced by adenosine infusion. A FFR value ≤0.8 indicates a functionally significant stenosis, and revascularization is associated with superior outcome (as compared to conservative therapy) [2]. However, FFR is currently not systematically assessed before revascularization related to technical reasons, procedure time or costs. Moreover, contra-indications to adenosine can limit FFR use which can be solved by application of adenosine-free functional indices. Results from a large clinical registry concerning attempted coronary interventions for intermediate stenoses (40–70% luminal narrowing) revealed that FFR is used only in 6.1% of the procedures prior to intervention [3].

These findings highlight the clinical need for alternative solutions of rapid physiologic assessment of coronary stenoses without the need for invasive introduction of pressure wires. The potential of quantitative flow ratio (QFR), based on 3-dimensional (3D) quantitative coronary angiography (QCA) and thrombolysis in myocardial infarction (TIMI) frame counting on hyperaemic images [adenosine-flow QFR (aQFR)], has been reported [4]. This technique has demonstrated good correlation and agreement with invasively measured FFR [4]. Moreover, Tu et al. recently showed that it is feasible to compute FFR using non-hyperaemic images for frame counting [contrast QFR (cQFR)] and using a fixed flow model which does not need frame counting [fixed QFR (fQFR)] [5]. However, some user interaction is needed for frame selection to assess the contrast transport time through the interrogated vessel. Furthermore, manual fine tuning of the 3D QCA coronary model and the reference contours of the coronary arteries is needed which may introduce inter-individual variability. Therefore, the current study assessed the accuracy and in particular the reproducibility of QFR computation using the aQFR, cQFR and fQFR flow model when compared with invasive FFR.

## Materials and methods

### Patients

Patients referred for invasive coronary angiography (ICA), who were eligible for FFR measurements were prospectively included. Patients presenting with acute coronary syndrome, with previous coronary artery bypass grafting or age <18 years were excluded. The study was approved by the ethical review committee of the Leiden University Medical Center and all patients provided written informed consent.

### Invasive coronary angiography and FFR measurement

ICA was performed according to standard protocols [6]. Angiographic projections were performed with mono- or biplane systems. At least two adequate contrast-filled angiographic projections with >25° apart (with minimum overlap) were acquired for QFR calculation. For FFR measurements, the pressure-wire (Brightwire 2; Volcano Corps, San Diego, CA, USA) was located distally to the lesion and maximal hyperaemia was induced by continuous intravenous infusion of adenosine (0.14 mg/kg/min). To enable QFR calculation by the aQFR model, one angiographic projection of the coronary artery of interest was acquired during hyperaemia at 30 frames/s. For the cQFR and fQFR models, no additional acquisitions were required during ICA and therefore all other projections were acquired with 15 frames/s.

### 3D QCA

The 3D QCA analyses were performed using validated software (QAngio XA 3D research edition 1.0, Medis Special BV, Leiden, The Netherlands) by 2 experienced observers. The 3D QCA measurements were performed as described previously [7]. In summary, two angiographic projections with angles >25° apart without overlap of the vessels were loaded. Properly contrast filled, end-diastolic frames of these two projections were selected. One to two anatomical landmarks were used as reference points in the two projections for automated correction of system distortions and possible patient motion between the two acquisitions [8]. Then, lumen contours were identified by automated 2D lumen edge detection algorithms and a reference contour of the coronary artery simulating the disease free luminal size of the artery was modelled. 3D reconstruction and modelling techniques were automatically performed. Lumen contours were manually adjusted where needed. Furthermore, lesion length, lumen area stenosis and diameter stenosis were calculated automatically with 3D-QCA.

### Quantitative flow ratio calculation

Details concerning the QFR calculation have been reported previously [4, 5]. Two observers calculated the QFR values according to the three methods, unaware of the pressure-wire FFR value and independently from each other. The location of the FFR pressure-wire was identified at the angiographic projections and the QFR values were measured at the same location. For each vessel the three different flow models were applied to a single 3D reconstruction of that vessel. The three different flow models were:

*Adenosine-flow QFR (aQFR)* using frame counts from adenosine induced hyperaemic images to measure the hyperaemic flow velocity.

*Contrast-flow QFR (cQFR)* using frame count analysis from regular (non-hyperaemic) angiographic projections to model hyperaemic flow velocity.

*Fixed-flow QFR (fQFR)* using a fixed empiric hyperaemic flow velocity derived from previous FFR studies [4]; no manual frame counting is needed.

### Flow rate by frame counting

**Fig. 1:**
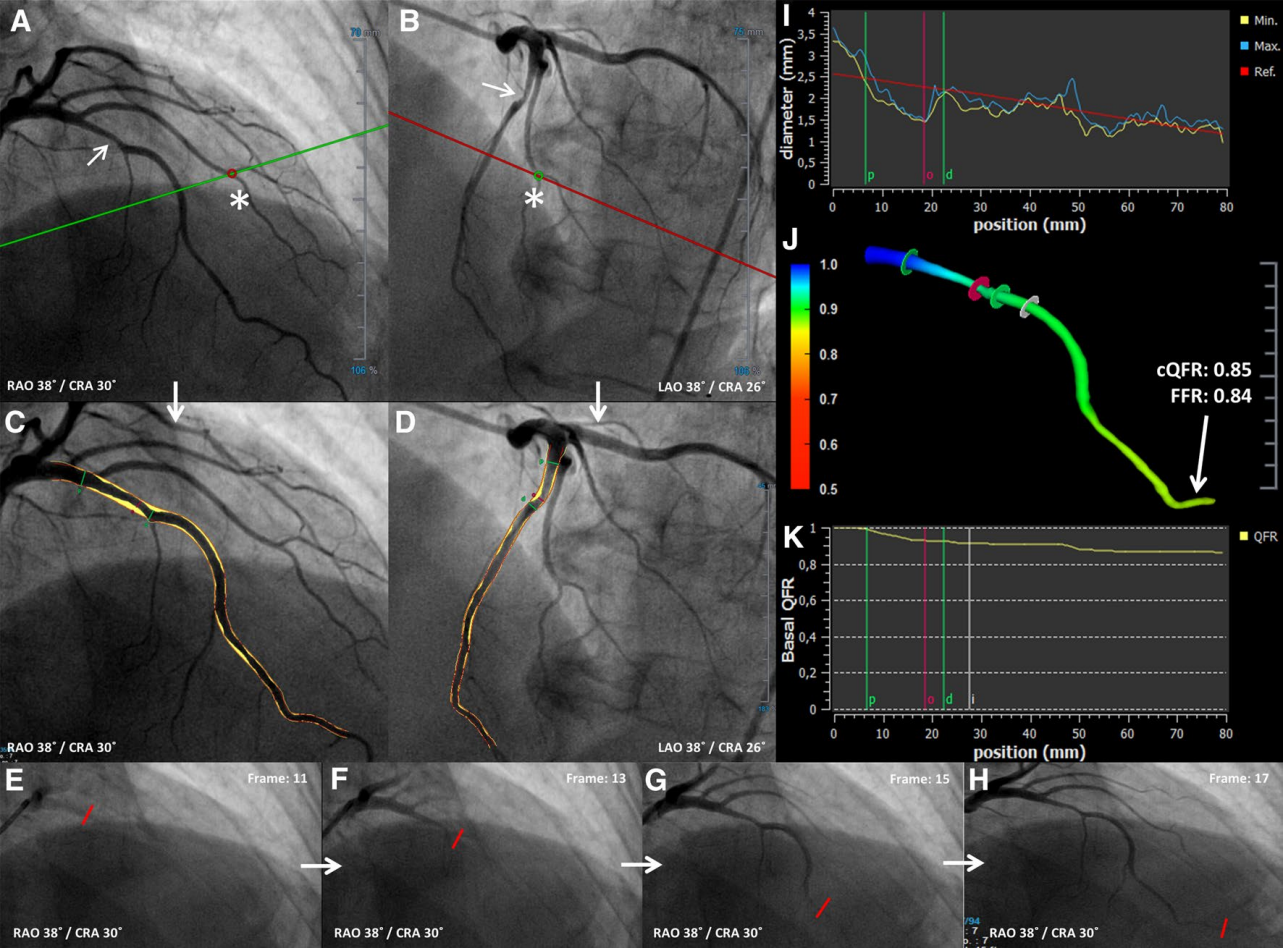
Example of computation of cQFR from 3D QCA and TIMI frame count. **a, b** 2 baseline, end-diastolic angiographic projections >25° apart in viewing angle. The *red* and *green* circle (*asterisk*) represent anatomical landmarks that serve as reference points in both projections for automated correction of angiographic system distortions. **c, d** After selection of baseline projections (**a, b**), the same projections (**c, d**) are used for automated lumen and vessel wall contour detection. *Yellow* represents coronary artery plaques (atherosclerosis).** e**–**h**: TIMI frame counting performed on one of the two baseline projections (**a, c**). The contrast bolus injection reached the proximal part of the quantified vessel segment at frame 11 (**e**). The distal part of the quantified segment was reached at frame 17 (**h**). The *red line* indicates the frontline of the contrast bolus. **i** The diameters of the vessel derived from the two projections. The *green lines* represent the proximal and distal part of the most severe coronary artery lesion and the purple line indicates the site of maximum stenosis severity. **j** 3D reconstruction of the coronary artery. The *colours* represent the decreasing QFR alongside the coronary artery. The cQFR at the most distal part of the analysed segment was 0.85; the invasively measured FFR was 0.84 at the same location. **k** 2D display of the pressure drop alongside the coronary artery

The transport time (number of frames) of the contrast bolus from the proximal to the distal part of the quantified segment of the coronary artery was assessed using the TIMI frame counting method [9]. An example of a cQFR computation is illustrated in Fig. 1.

### Statistical analysis

Continuous variables were depicted as mean ± SD. The one-sample T test was used to test whether the QFR and FFR values differed significantly from zero. First, agreement between the three different QFR techniques and FFR was assessed using Bland–Altman analyses and the Pearson’s correlation coefficient between the QFR and FFR values. Second, to assess inter-observer variability, Bland–Altman analyses were performed between the two observers. All statistical analyses were performed with the use of IBM SPSS Statistics software (version 20, IBM Corp, Armonk, New York, USA). A P value < 0.05 was considered statistically significant.

## Results

### Patients

A total of 17 patients (20 vessels) with intermediate coronary stenosis were included in the present study. Patient characteristics are presented in Table 1. One patient had a previous myocardial infarction and four patients had a prior PCI. No previous PCI was performed in the vessels in which QFR was calculated. Of the 20 vessels, five were not eligible for QFR calculation due to angiographic limitations (significant overlap of the vessel of interest with background vessels or non-eligible projections). Fifteen vessels (3 left circumflex and 12 left anterior descending coronary arteries) were included.

**Table 1:** Patient characteristics (n = 17)

Age, years	64 ± 11
BMI, kg/m^2^	27.7 ± 5.3
Male	71%
Prior PCI	24%
Prior myocardial infarction	6%
Cardiovascular risk factors	
Diabetes	6%
Hypertension	65%
Hypercholesterolemia	53%
Smoking	18%
Family history of CAD	12%

Values are mean ± SD or expressed as percentages

*BMI* body mass index, *CAD* coronary artery disease, *PCI* percutaneous coronary intervention

### Correlation and agreement between FFR and QFR per observer

**Fig. 2:**
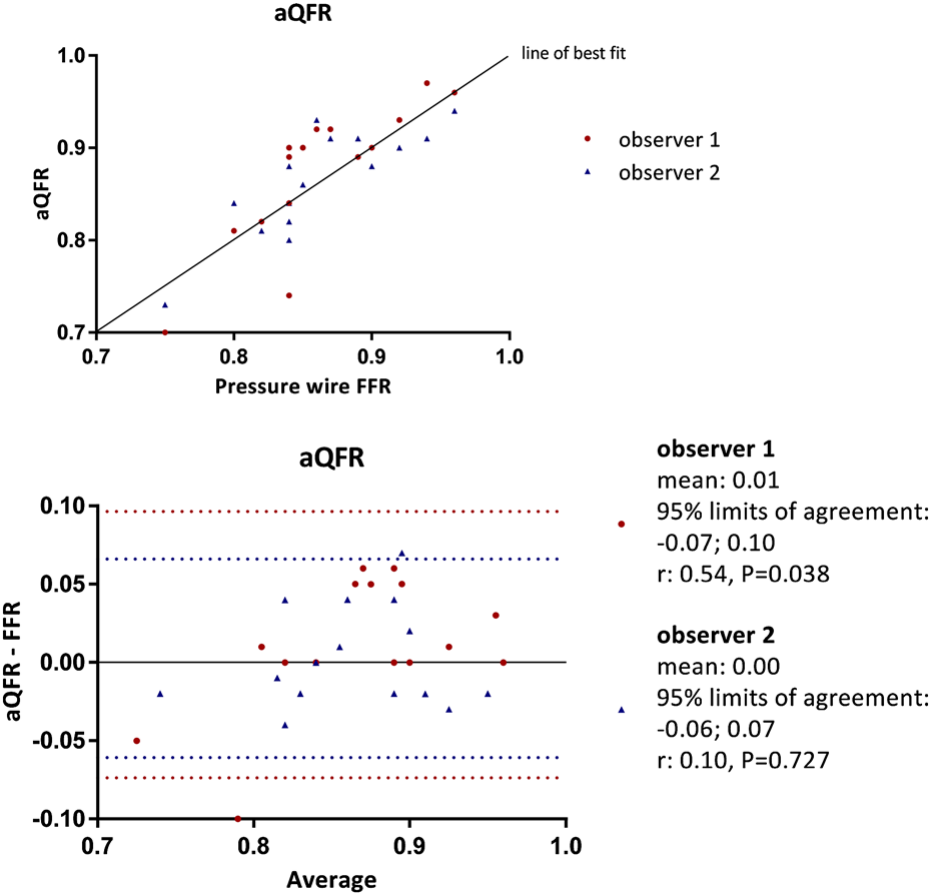
Correlation and Bland–Altman analysis between aQFR and FFR data

**Fig. 3:**
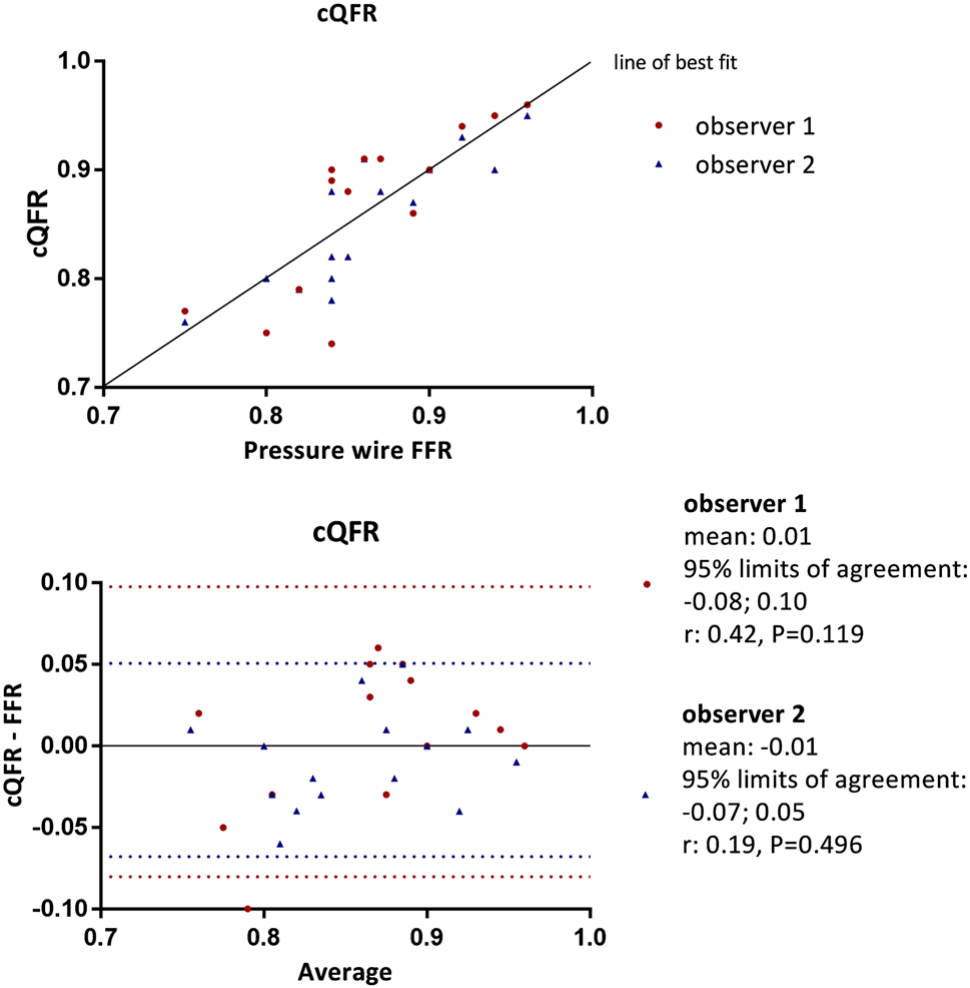
Correlation and Bland–Altman analysis between cQFR and FFR data

**Fig. 4:**
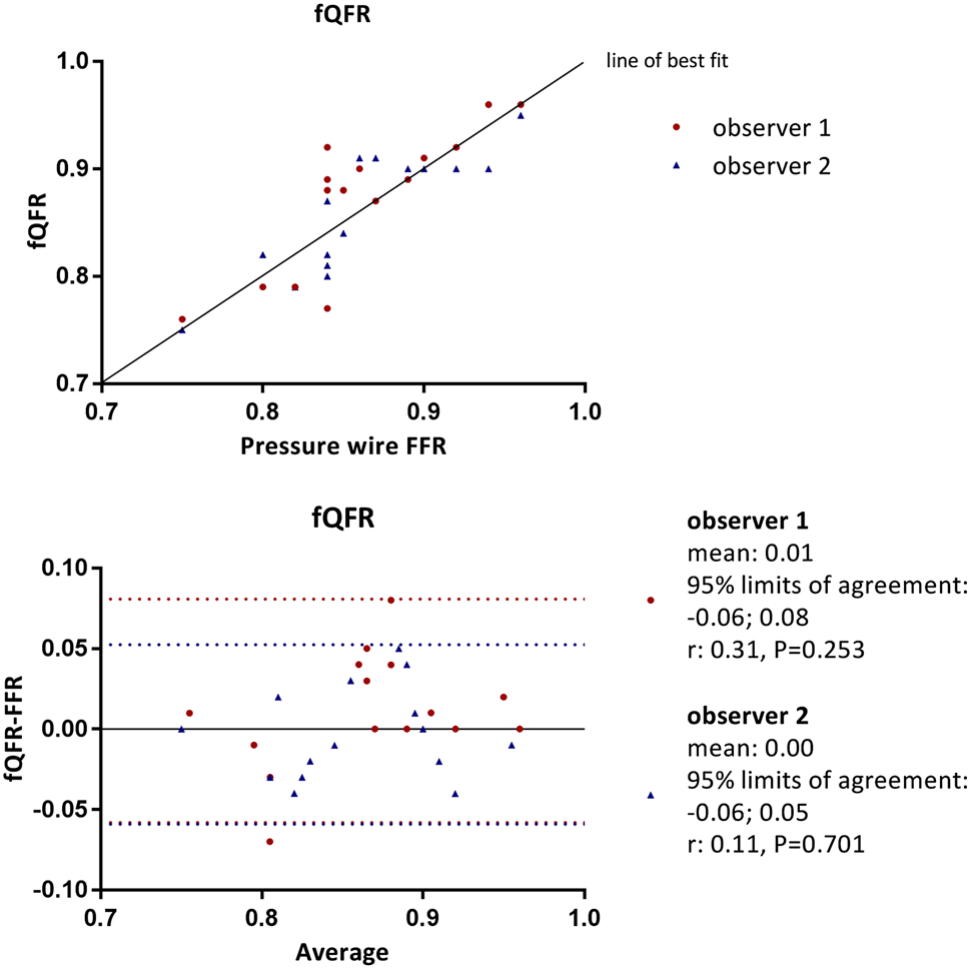
Correlation and Bland–Altman analysis between fQFR and FFR data

Table 2 shows the individual FFR data and the aQFR, cQFR and fQFR measurements of the 15 vessels. The mean difference between the aQFR, cQFR and fQFR measurements and the FFR data for observer 1 was: 0.01 ± 0.04, P = 0.329; 0.01 ± 0.05, P = 0.471; 0.01 ± 0.04, P = 0.236, respectively and for observer 2: 0.00 ± 0.03, P = 0.755; 0.01 ± 0.03, P = 0.285; 0.00 ± 0.03, P = 0.657, respectively. Hence, no systematic under- or overestimation of the QFR was observed when compared with the FFR data. Furthermore, Pearson’s correlations between FFR data and aQFR, cQFR and fQFR were good for observer 1: 0.84, P < 0.001; 0.78, P = 0.001; 0.839, P < 0.001, respectively and observer 2: 0.83, P < 0.001; 0.87, P < 0.001; 0.87, P < 0.001, respectively. Figures 2, 3 and 4 present scatter plots and Bland–Altman analyses of the aQFR, cQFR and fQFR measurements and the FFR data showing narrow 95% limits of agreement. No significant correlation was observed in the Bland–Altman plots, except for the aQFR for observer 1 (r: 0.54, P = 0.038). This indicates that the observed differences between QFR and FFR were not different for low and high FFR values.

**Table 2:** Difference between the wire-based FFR and the three QFR models

Number	Vessel	Wire-based FFR	Diameter stenosis^a^ (%)	Area stenosis^a^ (%)	Lesion length^a^ (mm)	QFR Observer 1			QFR Observer 2		
						Difference aQFR	Difference cQFR	Difference fQFR	Difference aQFR	Difference cQFR	Difference fQFR
1	LCX prox	0.96	31	43	16	0.00	0.00	0.00	−0.02	−0.01	−0.01
2	LAD prox	0.87	35	48	7	0.05	0.04	0.00	0.04	0.01	0.04
3	LAD mid	0.86	29	34	32	0.06	0.05	0.04	0.07	0.05	0.05
4	LAD mid	0.84	31	43	13	Na	0.06	0.08	Na	−0.02	−0.03
5	LAD mid	0.84	49	64	20	0.06	0.06	0.05	0.04	0.04	0.03
6	LCX	0.89	52	63	12	0.00	−0.03	0.00	0.02	−0.02	0.01
7	LAD mid	0.75	51	64	27	−0.05	0.02	0.01	−0.02	0.01	0.00
8	LAD mid	0.92	30	41	12	0.01	0.02	0.00	−0.02	0.01	−0.02
9	LAD mid	0.80	42	55	13	0.01	−0.05	−0.01	0.04	0.00	0.02
10	LCX	0.94	38	50	7	0.03	0.01	0.02	−0.03	−0.04	−0.04
11	LAD	0.84	45	58	14	−0.10	−0.10	−0.07	−0.02	−0.06	−0.02
12	LAD mid	0.82	33	45	15	0.00	−0.03	−0.03	−0.01	−0.03	−0.03
13	LAD mid	0.85	30	38	4	0.05	0.03	0.03	0.01	−0.03	−0.01
14	LAD mid	0.84	50	62	15	0.05	0.05	0.04	−0.04	−0.04	−0.04
15	LAD mid	0.90	34	48	26	0.00	0.00	0.01	−0.02	0.00	0.00
Average Standard deviation		0.86	38.7	50.4	15.4	0.01	0.01	0.01	0.00	−0.01	0.00
		0.05	8.6	10.0	7.7	0.04	0.05	0.04	0.03	0.03	0.03
P value						0.329	0.471	0.236	0.755	0.285	0.657

^a^Derived by 3D-QCA

### Inter-observer variability

**Fig. 5:**
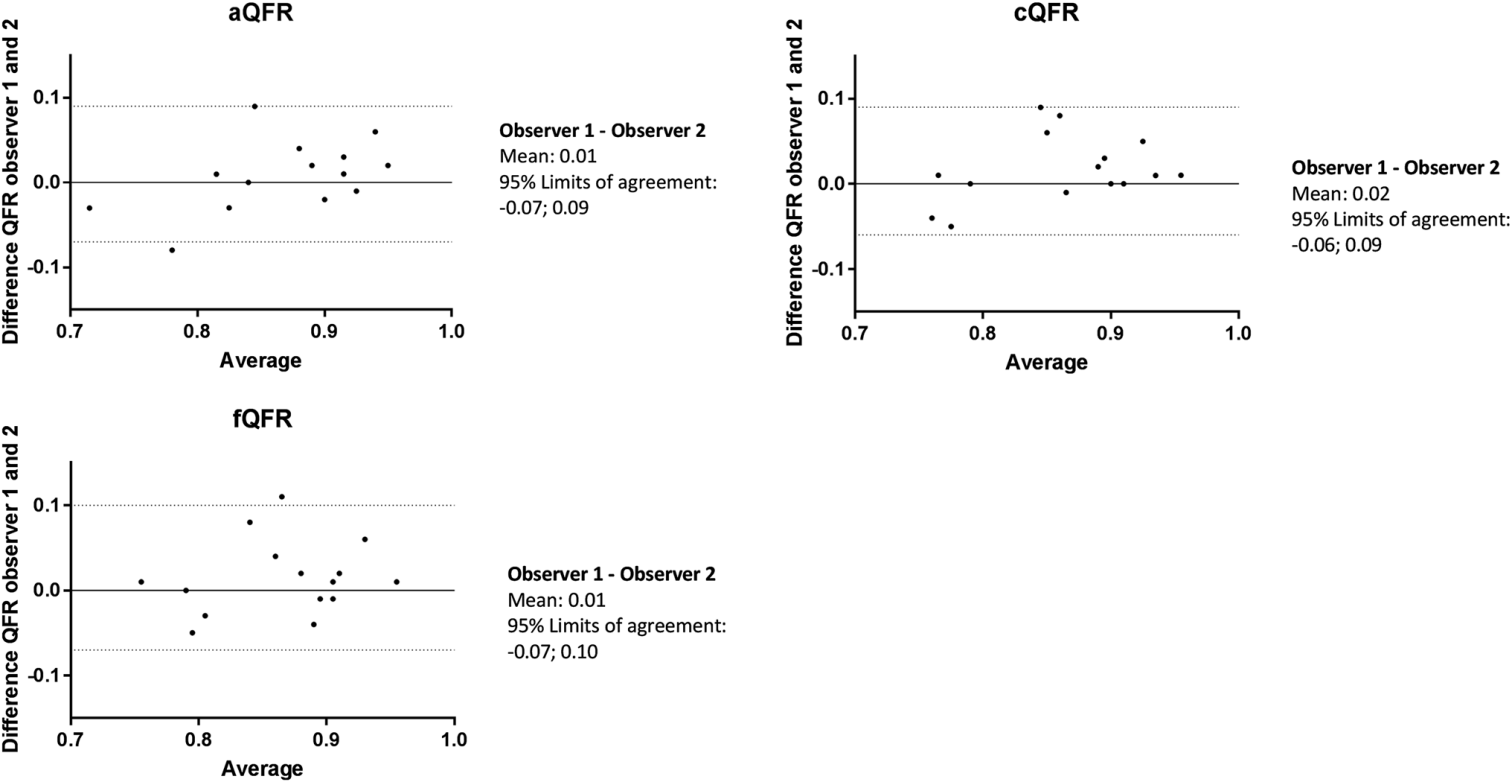
Inter-observer variability between the two observers

Figure 5 shows the Bland–Altman analyses between the two independent observers for the 3 QFR models. For the aQFR model, mean difference between the two observers was: 0.01 ± 0.04, for the cQFR model: 0.02 ± 0.04 and for the fQFR model: 0.01 ± 0.05.

## Discussion

This prospective study demonstrated, in a small group of patients, the accuracy and reproducibility of QFR calculation from 3D QCA using three different flow models: aQFR, cQFR and fQFR. All three methods showed good correlation and agreement with invasively measured FFR, and good agreement between the two observers.

Tu et al. were the first to report the diagnostic accuracy of the aQFR model in 77 vessels with intermediate coronary artery stenoses [4]. Compared with invasive FFR, the mean difference was 0.00 with a standard deviation of 0.06. However, the need for TIMI frame counting on hyperaemic images could be a limitation for its application into clinical practice, since adenosine infusion is needed. For this reason Tu et al. included the cQFR and fQFR (both models do not need hyperaemic images) in addition to the aQFR showing good agreement of these different methods with invasive FFR. However, besides highly accurate, new FFR computation techniques need to be reproducible when used by different analysts to become widely applied. Because some manual input is needed to refine the 3D QCA coronary tree model and for frame selection to assess contrast flow velocity, inter-observer variability may be introduced. This study adds new information to the previous work by Tu et al. [5] by demonstrating similarly good agreement between QFR-FFR and QFR–QFR by two observers who performed the analysis independently of each other. This implies that the QFR measurements are robust and reproducible. These three models can easily be implemented in the cardiac catheterization laboratory. Only two angiographic images with a different angle of at least 25° are required. The QFR can be calculated within 5 min (own experience) on site, which facilitates decision making regarding the need for coronary revascularization. Besides QFR calculation on site, it can also be measured off site after the acquisition of the angiogram, as performed in the current study. This approach is of interest for diagnostic cardiac catheterization laboratories without the possibility to perform interventional procedures. In the current study, five vessels were not eligible for QFR analysis because of vessel overlap. However, a newer version of the software contains an acquisition guide to determine the optimal viewing angles for a second good projection (which can then immediately be acquired during the angiography). This may reduce the number of non-eligible projections in the future.

### Limitations

The small sample size and the limited number of patients with low FFR values are limitations of the current study. Moreover, no right coronary artery was included in the current study. The fQFR employs a fixed flow model and does not require TIMI frame counting and ignores the influence of the coronary microvasculature circulation. Although the diagnostic accuracy of this method was not reduced in the present study, future research is needed to further validate the fQFR model in patients with increased microvascular resistance (e.g. diabetes or previous myocardial infarction).

## Conclusion

In this group of patients with intermediate coronary artery lesions, aQFR, cQFR and fQFR models for FFR calculation showed good agreement with invasively measured FFR and good inter-observer agreement. These results need further validation in larger studies with more heterogeneous patient populations.

### Impact on daily practice

The low rates of FFR measurement before PCI highlight the clinical need for alternative solutions of rapid physiologic assessment of coronary stenosis without the need for invasive introduction of pressure wires. In the current study, FFR was computed using the aQFR, cQFR and fQFR models which showed good correlation with invasively measured FFR and good agreement between two observers. The QFR may facilitate and increase physiological based revascularization.

## Abbreviations

CT: Computed tomography
3D: 3-dimensional
FFR: Fractional flow reserve
ICA: Invasive coronary angiography
QCA: Quantitative coronary angiography
QFR: Quantitative flow ratio
TIMI: Thrombolysis in myocardial infarction
